# Estradiol Represses the G_D3_ Synthase Gene *ST8SIA1* Expression in Human Breast Cancer Cells by Preventing NFκB Binding to *ST8SIA1* Promoter

**DOI:** 10.1371/journal.pone.0062559

**Published:** 2013-04-23

**Authors:** Marie Bobowski, Audrey Vincent, Agata Steenackers, Florent Colomb, Isabelle Van Seuningen, Sylvain Julien, Philippe Delannoy

**Affiliations:** 1 University Lille Nord de France, Lille, France; 2 Université des Sciences et Technologies de Lille (USTL), Unité de Glycobiologie Structurale et Fonctionnelle (UGSF), Villeneuve d’Ascq, France; 3 Centre National de la Recherche Scientifique (CNRS), Unité Mixte de Recherche (UMR) 8576, Villeneuve d’Ascq, France; 4 Institut National de la Santé et de la Recherche Médicale (INSERM), Jean Pierre Aubert Research Center, Lille, France; 5 Centre Hospitalier Régional et Universitaire de Lille, Lille, France; II Università di Napoli, Italy

## Abstract

Recent data have underlined a possible role of G_D3_ synthase (GD3S) and complex gangliosides in Estrogen Receptor (ER) negative breast cancer progression. Here, we describe the main transcript of the GD3S coding gene *ST8SIA1* expressed in breast tumors. We characterized the corresponding core promoter in Hs578T breast cancer cells and showed that estradiol decreases *ST8SIA1* mRNA expression in ER-positive MCF-7 cells and ERα-transfected ER-negative Hs578T cells. The activity of the core promoter sequence of *ST8SIA1* is also repressed by estradiol. The core promoter of *ST8SIA1* contains two putative Estrogen Response Elements (ERE) that were not found to be involved in the promoter activity pathway. However, NFκB was shown to be involved in *ST8SIA1* transcriptional activation and we demonstrated that estradiol prevents NFκB to bind to *ST8SIA1* core promoter in ERα expressing breast cancer cells by inhibiting p65 and p50 nucleus localization. The activation of NFκB pathway in ER-negative tumors, due to the absence of estradiol signaling, might explain the overexpression of G_D3_ synthase in this tumor subtype.

## Introduction

Gangliosides are glycosphingolipids carrying one or several sialic acid residues, and are essential compounds of the plasma membrane, by exposing their glycan moiety to the extracellular domain. They are enriched together with other phospholipids and cholesterol in lipid microdomains named “glycosynapses”, where they can modulate cell signaling, leading to changes in cellular phenotype [1], [2]. Glycosphingolipids from ganglio-series represent the main class of gangliosides and are usually classified in four series according to the presence of 0 to 3 sialic acid residues linked to lactosylceramide [3], [4].

While normal tissues usually express 0- and a-series gangliosides, gangliosides from b- and c-series are mostly expressed during embryogenesis and in the central nervous system in healthy adults, where they play a key role in cell-cell interaction, differentiation and growth [5], [6]. In parallel, complex gangliosides such as G_D3_, G_T3_ or G_D2_ have been found over-expressed in human tumors of neuroectoderm origin such as melanoma, glioblastoma and neuroblastoma [7]–[9]. They play a functional role in tumor growth and metastasis by mediating cell proliferation, migration, adhesion and angiogenesis [10]. Complex gangliosides have also been used as target molecules for cancer immunotherapy, such as G_D3_ in melanoma [11], [12] and G_D2_ in neuroblastoma [13], [14].

In normal breast tissues, complex gangliosides are absent or expressed at very low level. However G_D3_, 9-*O*-acetyl-G_D3_ and 9-*O*-acetyl-G_T3_ are oncofetal markers in invasive ductal breast carcinoma (IDC) [15]. Clinical studies have also shown that high expression of *ST8SIA1,* the gene coding the G_D3_ synthase (GD3S), is associated with Estrogen Receptor (ER) negativity and high histological grade of breast tumors [16], [17]. The GD3S is the only α2,8-sialyltransferase that synthesizes the disialoganglioside G_D3_ from its precursor G_M3_ [18], [19]. GD3S is therefore the key enzyme controlling the biosynthesis of complex gangliosides from b- and c- series.

In order to determine the role of complex gangliosides in breast cancer progression, we have previously induced GD3S over-expression in ER-negative MDA-MB-231 breast cancer cell line [20]. The resulting cellular model, MDA-MB-231 GD3S+, displayed a proliferative phenotype in absence of exogenous growth factor. This proliferative capacity of MDA-MB-231 GD3S+ clones directly proceeded from the constitutive activation of c-Met Tyrosine Kinase Receptor [21] and we recently showed that the ligand-independent activation of c-Met was due to the expression of G_D2_ ganglioside at the cell surface of GD3S+ clones [22]. Altogether, these data strongly suggest a possible role of GD3S and complex gangliosides in ER-negative breast cancer progression. Moreover, high G_D2_ expression was recently detected in breast cancer stem cells that was shown to be critical for mammosphere formation and tumor initiation [23]. Notably, GD3S but not G_M2_/G_D2_ synthase correlated with G_D2_ expression and GD3S knockdown reduced cancer stem cells properties and tumor formation.

To elucidate the molecular mechanisms leading to over-expression of GD3S in breast cancer, we have undertaken the study of the transcriptional regulation of the GD3S coding gene, *ST8SIA1,* in breast cancer cells. *ST8SIA1* is located on chromosome 12, in p12.1-p11.2 locus and consists in five coding exons spanning over 135 kbp [24]. Several reports have described the 5′-untranslated region (5′-UTR) of *ST8SIA1* in melanoma [24], [25], glioblastoma [26] and neuroblastoma [27] cell lines, showing a unique transcript with transcription start sites (TSS) located 400 to 650 bp upstream the initiation codon on the first exon. In this study, we described the main *ST8SIA1* transcript expressed in breast cancer tumors and cell lines and we characterized the core promoter of this gene. We also showed that estradiol repressed endogenous *ST8SIA1* mRNA expression as well as *ST8SIA1* core promoter activity by preventing NFκB binding in two human breast cancer cell lines expressing Estrogen Receptor alpha (ERα).

## Materials and Methods

### Breast Cancer Tumor Collection

20 tissue samples of IDC with ER-negative status (numbered 132 to 152) were provided by the Guy's and St Thomas's NHS foundation, Guy’s Hospital, London, United Kingdom. The NHS Research Ethics Committee (REC) approved the use of these tissues (ref: 07/H0804/131; HTA licence ref: 12121).

### Cell Culture

The human breast cancer cell line Hs578T [28] was kindly provided by Dr Van Slambrouck (New Mexico Institute of Mining and Technology, NM, USA). The human breast cancer cell line MCF-7 was obtained by the ATCC (Rockville, MD, USA). Both cell lines were routinely grown in Dulbecco’s Modified Eagle Medium (DMEM) with 4.5 g/L glucose, Ultraglutamine 1 supplemented with 10% fetal calf serum and 100 µg/mL penicillin-streptomycin (Lonza, Verviers, Belgium), at 37°C in an atmosphere of 5% CO_2_. When necessary, cells were grown for 48h in DMEM without phenol-red containing 10% charcoal-stripped serum (Invitrogen, Carlsbad, CA, USA) before treatment 24 or 12 h with 17-β-estradiol (Sigma Aldrich, Lyon, France) and/or Tamoxifen (TAM) (Sigma) and/or Tumor Necrosis Factor (TNF) (AbCys, Paris, France) or ethanol as vehicle.

### 5′-Rapid Amplification of cDNA Ends (5′-RACE)

The 5′-RACE system for Rapid Amplification of cDNA ends (Invitrogen) was used according to the protocol provided by the manufacturer. Initial reverse transcription was performed with the RT primer (annealing to the *ST8SIA1* sequence) using 4 µg of total RNA. After synthesis of the first strand cDNA, nested PCR was performed using Platinum® Taq DNA polymerase (Invitrogen). GSP1/Anchor primer and GSP2/AUAP primer pairs were used for first and second PCR, respectively. PCR products were size-separated by Agarose gel electrophoresis, subcloned into pCR2.1- TOPO vector (Invitrogen) and sequenced by Genoscreen (Lille, France).

### Plasmids Construction and Mutagenesis

*ESR1* open reading frame was amplified from MCF-7 cells cDNA with the primer pair ERαNhe1 and ERαKpn1 (Table 1). The PCR product was digested by Nhe1 and Kpn1 and cloned into the pcDNA3.1 expression vector (Invitrogen). The resulting plasmid was designed pcDNA-ERα. pCMV-p50 and pCMV-p65 vectors, expressing p50 and p65 NFκB subunits respectively, were previously described [29].

**Table 1:** Primers used for 5′-RACE, qPCR and plasmid constructions.

Primer	Sequence	
**RT primer** 1	5′-CACAGCCACTCTTCTT-3′	
**GSP1** 1	5′-CACCATTTCCCACCACCGCGATT-3′	
**GSP2** 1	5′-TTGCCTGTGGGAAGAGAGAGTAAGTTG-3′	
**Anchor primer** 2	5′-GGCCACGCGTCGACTAGTACGGGIIGGGIIGGGIIG-3′	
**AUAP** 2	5′-GGCCACGCGTCGACTAGTAC-3′	
**GD3SE4-E5 forward** 3	5′-TCCCAGCATAATTCGGCAAAGGTT-3′	
**GD3SE4-E5 reverse** 3	5′-ACCCTCAAAGATGGCTCTGTTCCT-3′	
**GD3SE1-E2 forward** 3	5′-AACGAGAAAGAGATCGTGCAG-3′	
**GD3SE1-E2 reverse** 3	5′-CCGTCATACCACATGCTCTTC-3′	
**PS2 forward** 3	5′-TAGACACTTCTGCAGGGATCTG-3′	
**PS2 reverse** 3	5′-GCAGTCAATCTGTGTTGTGAGC-3′	
**RPLP0 forward** 3	5′-GTGATGTGCAGCTGATCAAGACT-3′	
**RPLP0 reverse** 3	5′-GATGACCAGCCCAAAGGAGA-3′	
**HPRT forward** 3	5′-GCCAGACTTTGTTGGATTTG-3′	
**HPRT reverse** 3	5′-CTCTCATCTTAGGCTTTGTATTTTG-3′	
**ChIP-NFκB forward** 3	5′-AGAACAAGGGCACGAGTGAG-3′	
**ChIP-NFκB reverse** 3	5′-AGAAAAGAACTCGCTCTCATCAGT-3′	
**P1Nhe1 forward** 3	5′-CTCCCTGCTAGCTTTGCAGAAGAAAGAAAAACAGC-3′	
**P2Xho1 reverse** 3	5′-CAAATTCTCGAGCCCTCTGGACGTTTGTCG-3′	
**ERαNhe1 forward** 3	5′-GGGCTAGCCCATGACCATGACCCTCCA-3′	
**ERαKpn1 reverse** 3	5′-GGGGTACCATGCAGCAGGGATTATCTGA-3′	
**muERE1 forward** 4 **muERE1 reverse** 4 **muERE2 forward** 4 **muERE2 reverse** 4	5′- TGTCTGCCGTTATCTCCAAAGAACA**TA**GGCACGAGTGAGG3’5′- CCTCACTCGTGCC**TA**TGTTCTTTGGAGATAACGGCAGACA-3′ 5′-GGAGGGAGGGGGAGACC**AA**GATTTCCACCAATCCC-3′ 5′- GGGATTGGTGGAAATC**TT**GGTCTCCCCCTCCCTCC-3′	
**muERE1 reverse** 4	5′- CCTCACTCGTGCC**TA**TGTTCTTTGGAGATAACGGCAGACA-3′	
**muERE2 forward** 4	5′-GGAGGGAGGGGGAGACC**AA**GATTTCCACCAATCCC-3′	
**muERE2 reverse** 4	5′- GGGATTGGTGGAAATC**TT**GGTCTCCCCCTCCCTCC-3′	
**muNFκB forward** 4	5′-GGGAGGGAGG**AAA**AGACCTTGATTTCCACC-3′	
**muNFκB reverse** 4	5′-GGTGGAAATCAAGGTCT**TTT**CCTCCCTCCC	

1 Previously used by Kang *et al*. [25].

2 Provided by Invitrogen.

3 Designed using the NCBI primer design software (http://www.ncbi.nlm.nih.gov/tools/primer-blast/).

4 Designed using the QuickChange primer design software (http://labtools.stratagene.com/QC).

Restriction site sequences inserted for cloning are underlined and the mutated nucleotides in primers used for site directed mutagenesis are in bold.

Genomic DNA of Hs578T cells was prepared with Nucleospin Extract II kit (Macherey-Nagel, Düren, Germany) following manufacturer’s instructions. The genomic sequence located between −2307 bp and the ATG site was amplified by PCR using the primer pair P1Nhe1 and P2Xho1 (Table 1). The PCR product was cloned into the pCR2.1- TOPO vector (Invitrogen). The promoter sequence was then isolated by digestion of the vector using Nhe1 and Xho1 restriction sites. The purified fragment was sub-cloned into pGL3-Enhancer vector (Promega, Madison, USA) upstream of the firefly luciferase gene at Nhe1/Xho1 sites. The resulting plasmid was designed pGL3(−2307/+1). Truncated promoter constructs were generated by enzymatic digestions, ends blunting and ligation (Table 2). All plasmid constructs were sequenced to ensure the absence of mutation (Genoscreen, Lille, France).

**Table 2:** Reporter plasmid constructions.

Digestion	Position	Vector name
**NheI, XhoI**	−2307/+1	pGL3(−2307/+1)
**AleI, XhoI**	−1779/+1	pGL3(−1779/+1)
**HindIII, XhoI**	−1419/+1	pGL3(−1419/+1)
**SacII, XhoI**	−1117/+1	pGL3(−1117/+1)
**AlwNI, XhoI**	−923/+1	pGL3(−923/+1)
**BstxI, XhoI**	−565/+1	pGL3(−565/+1)
**FspI, XhoI**	−335/+1	pGL3(−335/+1)
**NheI, BstxI**	−2307/−565	pGL3(−2307/−565)
**AleI, BstxI**	−1779/−565	pGL3(−1779/−565)
**HindIII, Xho1**	−1419/−565	pGL3(−1419/−565)
**SacII, Xho1**	−1117/−565	pGL3(−1117/−565)
**AlwNI, Bstx**	−923/−565	pGL3(−923/−565)

Mutations with base substitutions at Estrogen Response Element (ERE) binding sites on pGL3(−923/−565) were obtained using a Quick Change mutagenesis kit (Stratagene, La Jolla, CA) according to the manufacturer’s protocol, using the oligonucleotide primers shown in Table 1. The plasmid mutations were verified by sequence analysis.

### RNA Extraction, cDNA Synthesis and Quantitative PCR

Total RNA from tumor samples or breast cancer cells was extracted using the Nucleospin RNA II (Macherey-Nagel). RNA quality was checked using the Agilent Bioanalyser 2100 (Agilent technologies, Santa Clara, CA, USA). Total RNA was reverse transcribed using Affinity script qPCR cDNA Synthesis kit (Agilent) according to the protocol provided by the manufacturer.

Primer sequences (Eurogentec, Seraing, Belgium) used for the PCR reactions are given in Table 1. Quantitative real-time Polymerase Chain Reaction (qPCR) was performed using the Mx3005p Quantitative System (Stratagene) as previously described [20]. Briefly, 40 cycles were performed according to the following program (94°C for 30 s, 60°C for 30 s, and extension at 72***°***C for 30 s).The analysis of amplification was performed using the Mx3005p software. For each primer pair, the specificity of the amplification was checked by recording the dissociation curves, visualizing the amplified products in Agarose-gel electrophoresis and sequencing of the products. *HPRT* or *RPLP0* genes were used to normalize the expression of transcripts of interest. Relative quantification was performed using the method described by Pfaffl, that takes in account the efficiency of each sequence amplification [30].

### Bioinformatic Analysis

*In silico* analysis of the promoter was performed with BLAST analysis of the human genome of the NCBI database. Multiple sequence alignments were performed with the Multalin program (http://multalin.toulouse.inra.fr/multalin/multalin.html). The core promoter sequence was analyzed with Matinspector 8.0 (www.genomatix.de) using TRANSAC matrices 8.4 [31] with “core similarity: 0.95″ and “matrice similarity : optimized”.

### Transient Transfection and Luciferase Assay

Hs578T cells (80% confluency) were transfected using lipofectamine (Invitrogen) according to the manufacturer’s instructions, with 1.5 µg of pGL3 construction and 20 ng of control *Renilla* plasmid in UltraMEM medium (Invitrogen). When necessary, 2 µg of pcDNA-ERα and 1 µg of pCMV-p50 and pCMV-p65 expression vectors were added to the transfection mix. After 6 h, the medium was replaced by fresh culture medium containing 10% FCS and further incubated for 48h. Cells were then washed with Phosphate Buffered Saline (PBS), lysed with Passive Lysis Buffer (PLB, Dual Luciferase Reporter Assay System, Promega, Madison, USA) and 20 µL of lysate were used for luciferase Reporter Assay System. Luminescence was measured with the Centro luminometer (Berthold Technologies, Bad Wildbad, Germany).

### Nuclear Protein Extraction and Western Blotting Analysis

ERα expressing Hs578T and MCF-7 cells transfected or not with pCMV-p50 and pCMV-p65 were lysed on ice in a hypotonic buffer (Hepes 10 mM, MgCl_2_ 1.5 mM, KCl 10 mM, pH 7.9) supplemented with 0.125% NP40 and protease cocktail inhibitors (Roche, Meylan, France). The lysate was centrifuged 5 min at 10,000 g. The pellet corresponding to the nuclear fraction was lysed with hypertonic buffer (Hepes 20 mM, MgCl_2_ 1.5 mM, EDTA 0.2 mM, NaCl 0.5 M, glycerol 25%, pH 7.9) supplemented with protease cocktail inhibitors on ice during 2 h. The protein concentration was determined with the Micro BCA™ Protein Assay Reagent kit (Pierce, Rockford, IL, USA). 40 µg of total proteins were boiled for 10 min in reducing Laemmli sample buffer and resolved by SDS-PAGE on 8% or 12% mini-gels (Bio-Rad, Richmond, USA). After transfer onto a nitrocellulose membrane (80 mA overnight), blocking was performed using Tris Buffer Saline (TBS) containing 0.05% Tween 20 and 5% (w/v) non-fat dried milk for 1 h at room temperature (RT). Incubations with anti-p65 (sc-7151x), anti-p50 (sc-1190x) mAbs (Santa Cruz Biotechnology Inc., Europe) and anti-histone H2B (07-371) mAb (Millipore, Billerica, USA) were performed 1 h at RT in TBS, 0.05% Tween 20 and 5% (w/v) non-fat dried milk. After washing, membranes were incubated 1 h at RT in TBS, 0.05% Tween 20 and 5% (w/v) non-fat dried milk of horseradish peroxydase conjugated to anti-mouse, anti-goat or anti-rabbit IgG. Membranes were finally washed 3 times for 10 min in TBS, 0.05% Tween 20 and detection was achieved using enhanced chemiluminescence (ECL+® advanceWestern blotting detection reagents, Amersham Biosciences, Little Chalfont, Buks, U.K.).

### Chromatin Immunoprecipitation (ChIP)

Hs578T-ERα and MCF-7 cells (1×10^6^ cells per antibody) were treated with 1% (v/v) formaldehyde for 10 min at room temperature and cross-links were quenched with glycine at a final concentration of 0.125 M for 5 min. Cells were rapidly rinsed with ice-cold D-PBS (Dulbecco-PBS) containing a cocktail of protease inhibitors (Roche), and scraped off and collected by centrifugation at 700 g for 5 min at 4°C, before being resuspended in lysis buffer (10 mM Hepes, pH 7.9, 10 mM KCl, 1.5 mM MgCl_2_ and 0.1% Nonidet P40) plus protease inhibitors and incubated for 10 min on ice. Chromatin was sheared with the Bioruptor system (Diagenode). The extracts were sonicated for 10 pulses of 30 s each with a 30 s rest between each pulse at 200 W at 4°C. After clearing by centrifugation at 10,000 g for 10 min at 4°C, the supernatant was fractionated and precipitated with either 3 µg of the specific antibody or normal goat IgGs (Upstate Biotechnology). An aliquot of the total supernatant was removed as input control. Immunoprecipitation was performed overnight on a rotating platform at 4°C, a mixture of Protein G and Protein A magnetic beads (Invitrogen) was then added and left for another 3 h as previously described [32]. Magnetic beads were collected and washed sequentially in Low Salt Immune Complex wash buffer (5 times), High Salt buffer (Upstate) and TE buffer [10 mM Tris/HCl (pH 8.0) and 1 mM EDTA]. The complexes were eluted with 210 µl of elution buffer [0.05 M Tris/HCl (pH 8.0), 0.01 M EDTA and 1% SDS] after a 15 min incubation at 65°C. Formaldehyde cross-links were reversed with 0.2 M NaCl at 65°C overnight. Chromatin-associated proteins were digested with Proteinase K at 37°C for 1 h and the DNA was purified with the Wizard® DNA Clean-up System (Promega). Samples were then subjected to qPCR analysis as described previously using ChIP-NFκB primers pair (Table 1). Percentage of input was determined using the Pfaffl method [30] and normalized to the IgG control which was set to 1.

## Results

### Identification of Human GD3S Transcripts in Breast Cancer Cells

**Figure 1:**
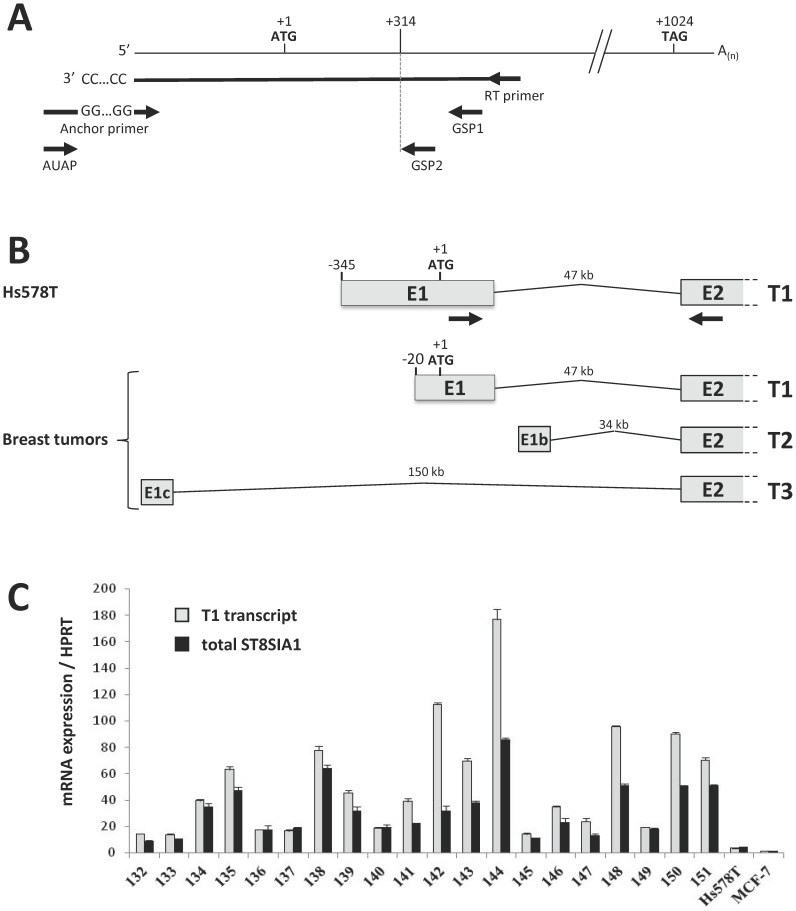
Identification of human GD3S transcripts in breast cancer tumors and Hs578T cells. (A) Schematic representation of the 5′-RACE strategy. First strand cDNA synthesis was performed with a *ST8SIA1* specific primer. cDNA was dC-tailed at 3′end and amplified by PCR using Anchor Primer and GSP1. Nested PCR was performed using AUAP and GSP2 primers. (B) Schematic representation of the main 5′-ends of GD3S transcripts expressed in Hs578T cells and breast cancer tumor samples (#137, #142 and #148). The size of intronic sequences between E2 and the different first exon are shown. Position of PCR primers used for specific amplification of T1 transcript is indicated by black arrows. (C) qPCR analysis of T1 transcript (grey) and total *ST8SIA1* (black) expression, related to *HPRT*, in 20 ER-negative IDC samples and 2 breast cancer cell lines (Hs578T and MCF-7).

The 5′-UTR of *ST8SIA1* transcripts was analyzed in Hs578T cell line (expressing *ST8SIA1* at higher level than others tested breast cancer cell lines) and 20 tumor samples of ER-negative IDC. The 5′-UTR of *ST8SIA1* transcripts were previously reported in melanoma, glioblastoma and neuroblastoma cell lines, showing a unique transcript, called T1, with several TSS within the first exon E1, from −400 to −650 bp upstream the ATG [24]–[27]. 5′-UTR amplification was performed for Hs578T cells and 3 tumor samples representative of the different levels of *ST8SIA1* expression (#137, #142 and #144) by 5′-RACE as described in the *Material and Methods* section (Fig. 1A). The sequencing of the resulting products indicated that *ST8SIA1* transcription also started within E1 exon, giving raise to T1 transcripts with different TSS between −345 and −20 bp upstream the ATG. Two minor transcripts (named T2 and T3) with alternative 5′-ends were also detected and located 34 and 150 kbp upstream E2 exon, respectively (Fig. 1B). The expression of T1 transcript was analyzed in 20 ER-negative IDC samples by qPCR and related to total *ST8SIA1* expression. As shown in Fig. 1C, the total expression of *ST8SIA1* was higher in tumor samples compared to Hs578T cells and T1 accounted for 50 to 100% of the total transcripts, making it the main *ST8SIA1* transcript across this series of tumor samples.

### Promoter Activity of the 5′-flanking Region Upstream the GD3S T1 Transcript

**Figure 2:**
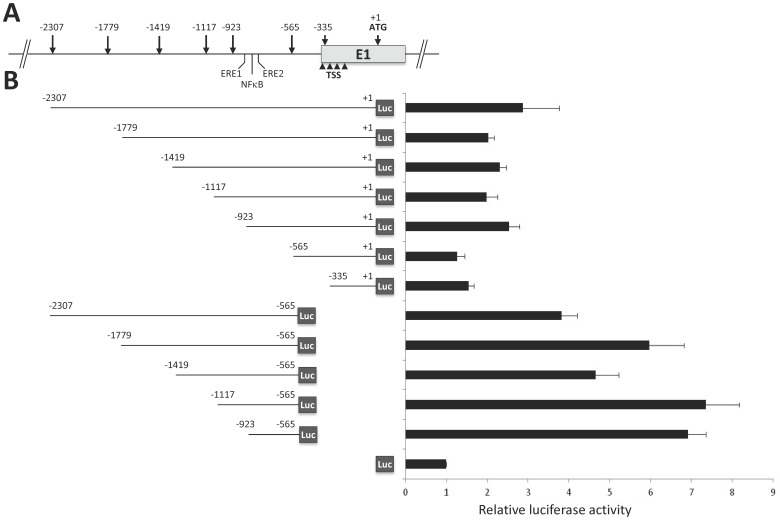
Promoter activity of the 5′ flanking region of GD3S T1 transcript in Hs578T cells. (A) Location of the restriction sites used to generate the different deletions of the genomic sequence between −2307 and the ATG site (+1) in E1 exon. The positions of EREs and NFκB binding site in the core promoter are indicated. Arrowheads show the position of TSS from −345 to −20 bp upstream the ATG. (B) On the left, schematic representation of the different constructs inserted in pGL3basic upstream the luciferase gene. Luc indicates the Firefly luciferase coding sequence. On the right, luminescence detected in luciferase assays. Transfection efficiencies were normalized with the co-transfected plasmid expressing *Renilla* luciferase and luciferase activities are expressed compared to empty pGL3basic activity. The data are means +/− S.D. of n ≥3 experiments.

To determine the core promoter sequence of the T1 transcript, the genomic sequence located between −2307 bp and the ATG site in E1 was cloned into the pGL3basic upstream the luciferase gene and named pGL3(−2307/+1). This plasmid and the 5'- or 3′-deleted constructs, were transfected into Hs578T cells for luciferase assays. The results presented in Fig. 2 showed a 2.9-fold increase of luciferase activity for the full length plasmid pGL3(−2307/+1) compared to pGL3basic used as baseline control. By comparison, all constructs lacking the −565/+1 region showed increased activities with maximal promoter activities for pGL3(−1117/−565) and pGL3(−923/−565) constructs (7.4- and 7.0-fold, respectively). In parallel, 5′ truncations of various length induced decreased luciferase activity with pGL3(−565/+1) and pGL3(−335/+1) constructs showing almost no activity compared to pGL3basic (1.2- and 1.5-fold, respectively). Together, these data suggest the existence of a core promoter region of GD3S within the sequence −923/−565 and a negative regulation region within −565/+1.

### Bioinformatics’ Analysis of T1 Core Promoter Region

The putative core promoter sequence −923/−565 was analyzed with Matinspector software with “core similarity: 0.95″ and “matrice similarity: optimized”. This analysis did not reveal any canonical TATA or CAAT boxes as previously reported by others [24]. However, a large number of putative binding sites for general (e.g. SP1) and specific transcription factors were retrieved. Positions of putative transcription factors related to breast cancer are presented in Table 3. Notably, two putative Estrogen Response Element (ERE) were found at position −867/−844 (ERE1) and −783/−760 (ERE2),

**Table 3:** Predicted transcription factors binding sites in −923/−565 core promoter and their relevance in breast cancer.

Transcription factor	Core sequence	Position	Strand	Involvement in breast cancer
**WT1**(Wilm’s tumor 1)	CGGGTGGGAGGG	−914/−898−793/−777−783/−767	+−	Involved in proliferation and differentiation [47]. Over-expressed in ER-negative tumors [48]
**c-Myb**	CAACTAAC	−906/−892−693/−679	−+	Oncogene. Stimulates cell proliferation [49]
**NFκB**	GGGA	−777/−762	+	Downregulated by ER signaling [43]
**ERE** (Estrogen Response Element)	AAGG	−867/−844−783/−760	+−	Regulation of estrogen-responsive genes [50]
**E2F2**	GCGC	−849/−833−710/−694	+/−+/−	Regulated by estradiol [51]
**KLF15** (Krüppel Like Factor 15)	GGGG	−789/773	+	Regulates estradiol-induced proliferation [52]
**NFAT** (Nuclear Factor of Activated T cells)	GGAA	−770/−752−589/−571	++/−	Pro-invasive and pro-migratory [53]
**ETS-1**	GGAA	−723/−703	+	Associated to invasive phenotype [54]

### Estradiol Represses Endogenous ST8SIA1 Transcripts in ER-positive MCF-7 and in ER-negative ERα-transfected Hs578T Cells

**Figure 3:**
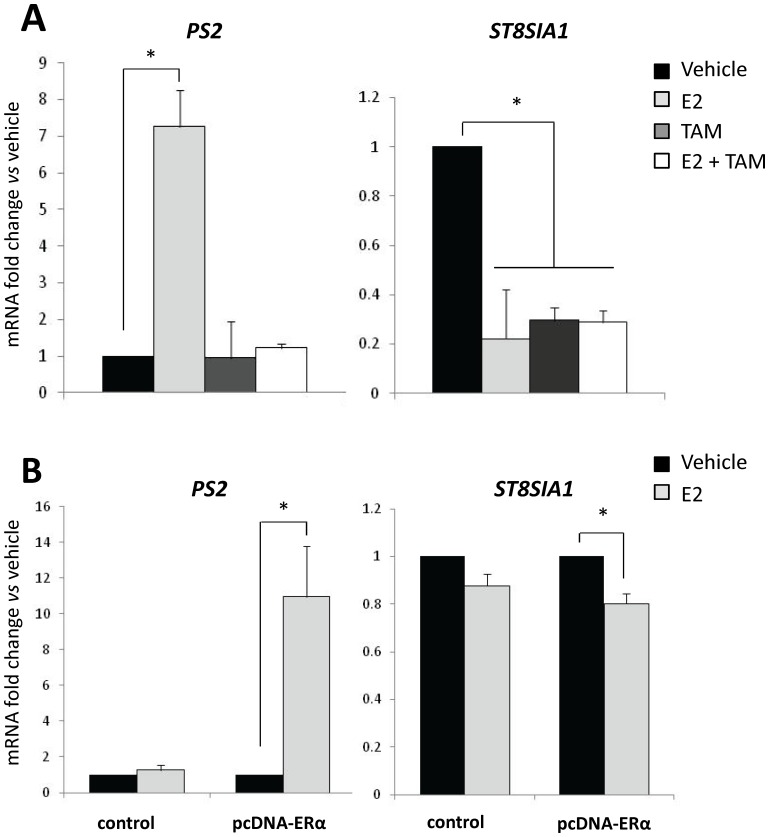
Estradiol represses *ST8SIA1* mRNA expression in ER-positive MCF-7 and in ER-negative Hs578T expressing ERα. (A) Effect of estradiol and Tamoxifen on GD3S mRNA expression in MCF-7 cells. After 48h of culture in steroid-free medium, MCF-7 cells were treated with 10^−10^ M estradiol and/or 10^−6^ M TAM for 24h. (B) Effect of estradiol on GD3S mRNA expression in Hs578T cells transfected with ERα coding vector. After 48h of culture in steroid-free medium, Hs578T cells were transfected with pcDNA-ERα or pcDNA empty vector (control). 24h after transfection, cells were treated for 24h with 10^−10^ M estradiol. For (A) and (B), *ST8SIA1* or *PS2* (positive control) mRNA expression was determined by qPCR. Results were normalized to the expression of *RPLP0* and reported to the expression of *ST8SIA1* or *PS2* in cells treated with vehicle (0.1% ethanol). Data are means +/− SD of n ≥3 experiments. * p<0.05 *vs*. untreated (vehicle).

Given that *ST8SIA1* is over-expressed in ER-negative breast tumors [16] and that its core promoter contains two putative sites for ERα binding, we investigated the effect of estradiol on *ST8SIA1* mRNA expression in breast cancer cell lines MCF-7 and Hs578T. *ST8SIA1* expression was analyzed by qPCR in ER-positive MCF-7 cells treated with estradiol (10^−10^ M) and/or Tamoxifen (10^−6^ M) for 24h. *PS2*/*TFF1* (Trefoil Factor 1) gene, known to be up-regulated by estradiol [33] was similarly analyzed to control the estradiol responsiveness of the treated cells. As shown in Fig. 3A, estradiol expectedly increased *PS2* expression while *ST8SIA1* expression decreased significantly (about 4.5 fold). TAM treatment abolished the increased PS2 expression but has no antagonistic effect on estradiol-mediated GD3S mRNA repression. Similar experiment was performed in ER-negative Hs578T cells transfected or not with the pcDNA-ERα vector 24h before estradiol treatment. Estradiol had no significant effect on the expression of *PS2* or *ST8SIA1* in mock-transfected Hs578T cells (Fig. 3B). In contrast, in Hs578T exogenously over-expressing ERα, estradiol induced an increase of *PS2* expression as well as a slight but significant decrease of *ST8SIA1* expression. Taken together, our results demonstrate that estradiol represses *ST8SIA1* expression in an ERα dependent manner.

### The Core Promoter Activity is Inhibited by Estradiol through an ERE-independent Manner Involving NFκB Transcription Factor

**Figure 4:**
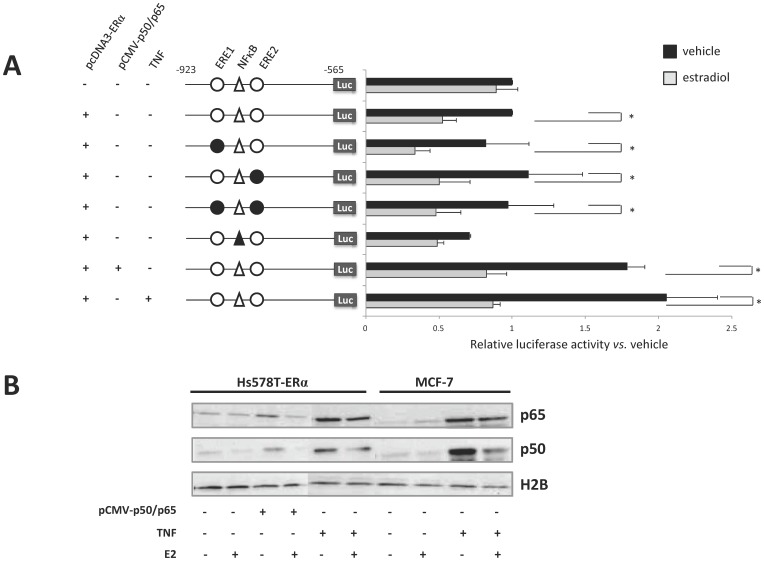
Estradiol-mediated repression of *ST8SIA1* promoter activity do not involve ERE element but NFκB transcription factor. (A) Effect of mutations of ERE and NFκB putative sites on −923/−565 core promoter activity. After 48h of culture in steroid-free medium, Hs578T were transfected with pGL3(−923/−565), either native or mutated on ERE binding sites, pcDNA-ERα and/or pCMV-p65 and pCMV-p50. Transfection with empty expression vectors was used as negative controls. The following day, cells were treated for 12 h with 10^−10^ M estradiol and/or 40 ng/mL TNF. On the left: schematic representation of the sequence transfected in Hs578T cells. Black circles indicate the mutated ERE sequences and black triangle indicates the mutated NFκB site. On the right: relative luciferase activity of the core promoter in Hs578T cells treated or not with 10^−10^ M estradiol. Transfection efficiencies were normalized with the co-transfected plasmid expressing *Renilla* luciferase and reported to the luciferase activity in cells transfected with native pGL3(−923/−565) treated with vehicle. Vehicle: 0.1% ethanol. Each bar represents the mean +/− S.D. of n ≥3 experiments. *: p<0.05. (B) Effect of estradiol on p50 and p65 NFκB subunits nuclear expression in ERα expressing Hs578T and MCF-7 cells. Nuclear proteins extracted from ERα-transfected Hs578T and MCF-7 cells treated as previously described in A were used for immunoblotting with anti-p50 or anti-p65 mAbs. Histone H2B expression was used as a loading control.

To demonstrate the role of EREs found in the core promoter in the estradiol-mediated *ST8SIA1* regulation, we analyzed the activity of the promoter sequence −923/−565 in estradiol treated Hs578T cells transfected or not with pcDNA-ERα. As shown in Fig. 4A, estradiol had no significant effect on the core promoter activity in mock-transfected Hs578T cells (control). In parallel, ERα expression allowed a significant decrease of the luciferase activity (50%) in estradiol treated cells. A similar fold of repression was obtained for the pGL3(−1117/−565) construct (data not shown). However, site-directed mutagenesis of either one or both putative ERE on pGL3(−923/−565) construct did not suppress the repressive action of estradiol (Fig. 4A). This result suggests that the ERE sites are not involved in ER signaling and that estradiol exerts an indirect effect on the *ST8SIA1* promoter activity. Lee and coworkers described a functional NFκB binding site on *ST8SIA1* promoter in melanoma cells [25], [27] that we also confirmed by bioinformatic analysis at position −777/−762 (Table 3). As shown in Fig. 4A, the mutation of NFκB binding site led to a significant decrease of luciferase activity (30%), demonstrating NFκB to be involved in *ST8SIA1* promoter activity in breast cancer cells. Notably, directed mutagenesis of others predicted transcription factors binding sites described in Table 3 have no effect on the activity of the core promoter (data not shown). Moreover, p50 and p65 expression vectors transfection or TNF treatment, both inducing an increase of p50 and p65 NFκB subunits in the nucleus (Fig. 4B), resulted in a 2-fold increase of luciferase activity (Fig. 4A). Furthermore, we showed that estradiol inhibited the NFκB-mediated increase of *ST8SIA1* promoter activity in Hs578T-ERα cells (Fig. 4A). This could be explained by the inhibitory effect of estradiol on p50 and p65 nuclear localization (Fig. 4B). Altogether, these data show the role of NFκB in transcriptional activation of *ST8SIA1* promoter in breast cancer cells and suggest that estradiol inhibits NFκB-mediated activation by preventing p50 and p65 NFκB subunits translocation into the nucleus.

### Estradiol Decreased Endogenous NFκB Binding to ST8SIA1 Promoter in ERα-Expressing Breast Cancer Cell Lines

**Figure 5:**
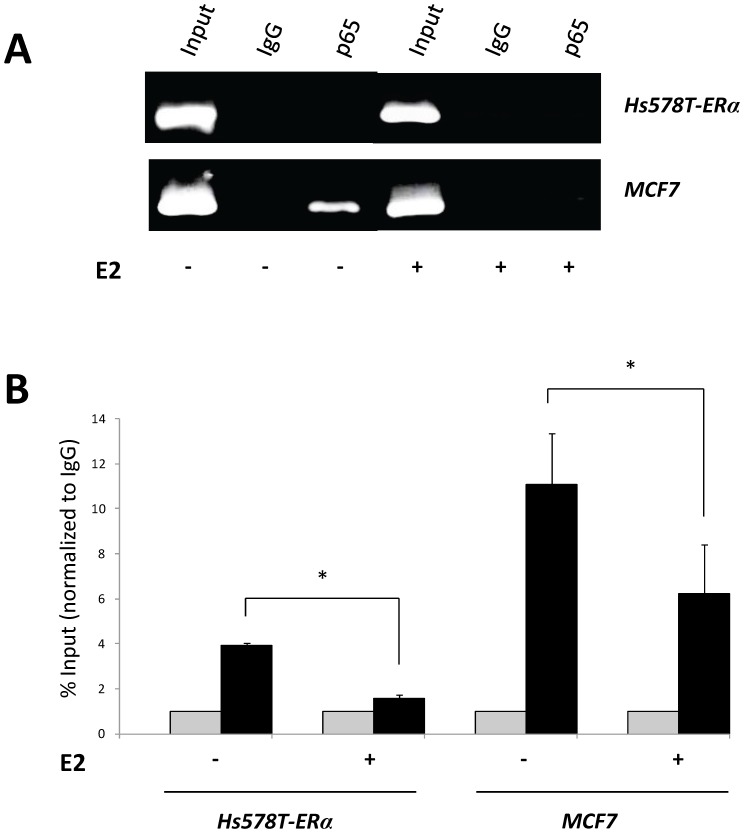
*In vivo* binding of p65 subunit to *ST8SIA1* promoter by Chromatin Immunoprecipitation in breast cancer cells. ChIP were performed as described in the Material and Methods from ERα-expressing Hs578T cells and MCF-7 cells treated 12 h with 40 ng/mL TNF in presence or in absence of 10^−10^ nM estradiol. PCRs were carried out with specific pairs of primers covering the NFκB binding site (−773/−769). PCR products were analyzed on 2% Agarose gel (A) or *via* qPCR (B). Bars indicate percent enrichment compared to the input DNA and were normalized to the goat isotype control. Each bar represents the mean +/− S.D. of n ≥2 experiments. *: p<0.05.

**Figure 6:**
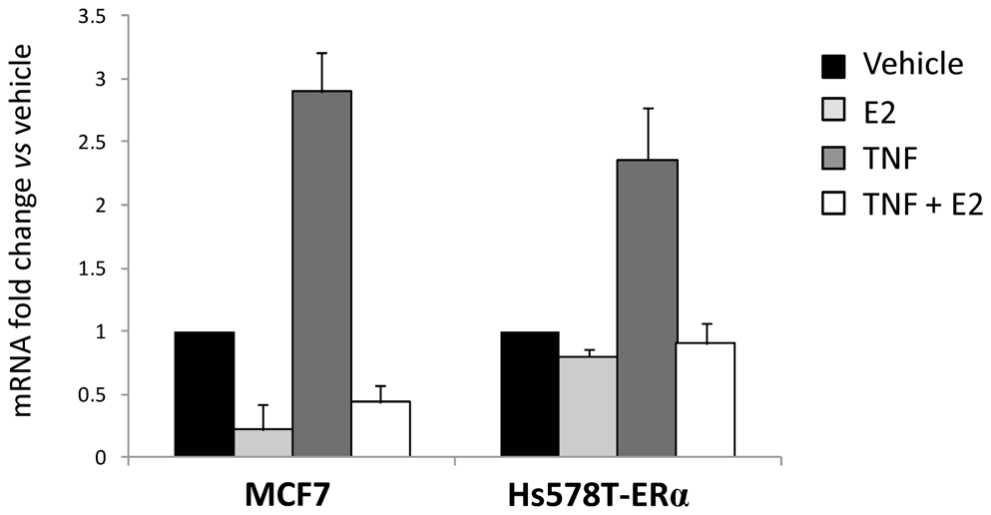
Estradiol reverses the TNF-mediated increase of *ST8SIA1* mRNA expression in ER-positive MCF-7 and in ER-negative Hs578T expressing ERα. ERα-expressing Hs578T and MCF-7 cells were treated with 10^−10^ M estradiol and/or 40 ng/mL TNF for 12h. *ST8SIA1* mRNA expression was determined by qPCR. Results were normalized to the expression of *RPLP0* and reported to the expression of *ST8SIA1* in cells treated with vehicle (0.1% ethanol). Data are means +/− SD of n ≥3 experiments. *: p<0.05 *vs*. untreated (vehicle).

To confirm that NFκB directly acts on *ST8SIA1* promoter, ChIP experiments were performed using Hs578T expressing ERα and MCF-7 cells treated with TNF, in absence or in presence of estradiol (Fig. 5). We found that the region −868/−665 of the *ST8SIA1* promoter, encompassing the −777/−762 NFκB binding site, was enriched for p65 over the negative control IgG both in Hs578T-ERα and MCF-7 cell lines, showing that p65 directly binds this promoter region in these cells. Estradiol treatment led to a significant decrease of p65 binding in both Hs578T-ERα and MCF-7 cell lines. As shown in Fig. 6, the loss of NFκB binding on *ST8SIA1* promoter is associated with a decrease of endogenous *ST8SIA1* mRNA expression in both MCF-7 and Hs578T-ERα cells. Furthermore, confirming the results obtained with the core-promoter constructs, TNF treatment of MCF-7 and Hs578T-ERα cells led to a 2.9- and 2.4-fold increase of *ST8SIA1* mRNA expression, respectively, and estradiol treatment reverted this effect.

## Discussion

In this paper, we determined that T1 is the major transcript of GD3S expressed in breast tumor tissues and we characterized the core promoter essential for transcription of GD3S in human breast cancer cells. In breast tumor tissues, the 5′-UTR of T1 transcript is shorter but similar to the one found in other cell lines [24]–[27]. Furthermore, we newly described minor transcripts as T2 and T3 with alternative first exon that lack initiation codon. Similar aberrant non-coding transcripts have recently been described in tumors and have been suggested to play functional roles in cancer progression and metastasis [34]. Interestingly, the core promoter we identified in breast cancer cells, between −923 and −565 upstream the initiation codon, overlaps with the core promoter found in melanoma (−833/−519) [24] and neuroblastoma (−1190/−690) [27] but not with the one found in glioblastoma (−1330/−1190) [26], suggesting a tissue-specific regulation of *ST8SIA1.*

In the present study, we show an ERα-mediated estradiol down-regulation of both endogenous GD3S mRNA and core promoter activity in breast cancer cells. This result fits well with a microarray analysis showing an inverse correlation between *ST8SIA1* and *ESR1* (coding ERα receptor) gene expression in invasive breast cancer primary tumors [16], [17]. We therefore propose that high expression of GD3S in ER-negative tumors, due to the loss of ERα signaling, could increase complex gangliosides expression at the cell surface, and through this possibly enhance the aggressiveness of this tumor subtype [21], [23].

Transcriptional regulation by steroid hormones has been showed for several sialyltransferases. For instance, ST3Gal III and ST6Gal I were demonstrated to be up-regulated and down-regulated respectively by estradiol in MCF-7 breast cancer cells [35]. Testosterone was also shown to up-regulate ST3Gal II expression, through epigenetic regulation involving NFκB, in prostate cancer cells [36].

TAM is an estradiol antagonist used to treat ER-positive breast cancer patients. It plays an active role in inhibition of breast cancer cells proliferation through repression of ERα responsive genes normally involved in cell proliferation [37]. Although TAM treatment of Hs578T cells efficiently prevented the expression of *PS2*, a well characterized estradiol responsive gene, it did not compete with estradiol-induced *ST8SIA1* repression. Reassuringly, our results suggest that TAM treatment of breast cancer patients is unlikely to induce the expression of possibly deleterious complex gangliosides *via ST8SIA1* induction in ER-positive breast cancer tumors.

Although bioinformatics analysis indicate two ERE on the core promoter, site mutagenesis of these predicted EREs in Hs578T cells failed to confirm their *cis*-regulatory function in *ST8SIA1* transcription. However, it was reported that estradiol-mediated down-regulation of gene expression can be classified in two groups according to the kinetic of their response to the hormone [38], [39]. Early-down-regulated genes are often primary targets of estrogen receptor, while late-down-regulated genes require secondary factors for transcription [38]. In our cells, *ST8SIA1* down-regulation was only observed after 8h of estradiol treatment (data not shown) suggesting *ST8SIA1* to be indirectly regulated by estradiol (i.e. without direct binding of ERα to *ST8SIA1* promoter).

**Figure 7:**
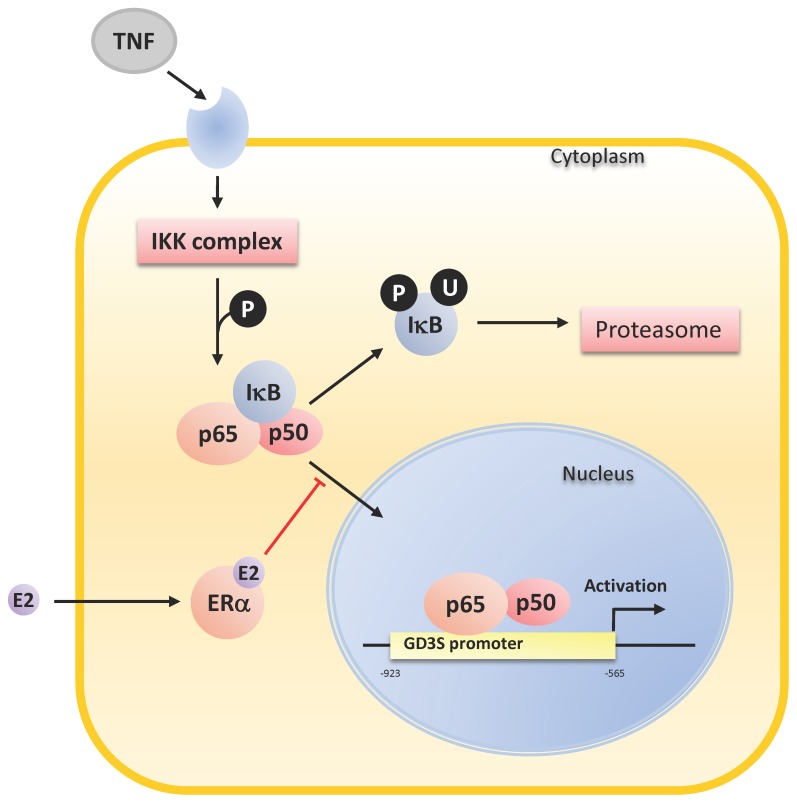
Hypothetical mechanism of GD3S repression by estradiol. GD3S gene (*ST8SIA1*) is activated by the canonical NFκB pathway after TNF stimulation. In ER-positive breast cancer cells, GD3S expression is repressed by estradiol (E2)-ERα complex. Repression of NFκB transport to the nucleus could be achieved via the activation of PI3K [44].

A functional NFκB binding site at −777/−762 pb upstream the ATG was shown to be essential for *ST8SIA1* transcription in melanoma cells [25], [27]. NFκB is a transcription factor frequently activated in tumors that is involved in tumor growth, progression and resistance to chemotherapy [40]. In particular, activated NFκB is predominantly detected in ER-negative *vs*. ER-positive breast tumors [41], [42]. Accordingly, several studies have shown that ERα can inhibit NFκB activity in an estradiol-dependent manner in various cell lines [43]. For example, estradiol was shown to prevent p65 activation and to inhibit its intracellular transport to the nucleus *via* the activation of PI3K. This effect is mediated by ERα and selectively activated in macrophages to prevent the inflammatory response [44]. It was also shown that estradiol inhibits TNF-induced NFκB activation in MCF-7 cells [45]. Here, we show that NFκB is involved in transcriptional activation of GD3S in both Hs578T-ERα and MCF-7 breast cancer cells and that estradiol represses GD3S expression by inhibiting p65 and p50 nucleus transport (Fig. 7). This could explain the higher expression of GD3S in ER-negative breast cancer cells and tumors, in which the effect of NFκB cannot be repressed by estradiol, leading to a higher amount of complex gangliosides that reinforce proliferative capacity of the tumor. Delineating the molecular mechanisms by which estradiol represses GD3S in breast cancer cells could provide new targets to inhibit complex gangliosides synthesis and potentially hamper ER-negative breast tumors aggressiveness. Potential therapeutic strategy targeting NFκB would prevent complex gangliosides expression in ER-negative breast tumors. Drugs that inhibit NFκB signaling have been identified and are currently undergoing clinical trials in combination with standard anti-tumor agents to achieve a better treatment of tumors and an increase in survival [46].
